# Geographic Variation in Note Types of Alarm Calls in Japanese Tits (*Parus minor*)

**DOI:** 10.3390/ani12182342

**Published:** 2022-09-08

**Authors:** Li Zhang, Jiangping Yu, Chao Shen, Dake Yin, Longru Jin, Wei Liang, Haitao Wang

**Affiliations:** 1 Jilin Engineering Laboratory for Avian Ecology and Conservation Genetics, School of Life Sciences, Northeast Normal University, Changchun 130024, China; 2 Ministry of Education Key Laboratory for Ecology of Tropical Islands, Key Laboratory of Tropical Animal and Plant Ecology of Hainan Province, College of Life Sciences, Hainan Normal University, Haikou 571158, China; 3 Jilin Provincial Key Laboratory of Animal Resource Conservation and Utilization, Northeast Normal University, Changchun 130024, China

**Keywords:** note, bird alarm calls, geographic variation, Japanese tits

## Abstract

**Simple Summary:**

Divergence in acoustic signal systems might play a central role in speciation. Alarm calls are part of the acoustic signal system, which can transmit information about impending threats to group members and relatives. This study focuses on geographic variation in the note types of alarm calls in Japanese tits, a small songbird species distributed broadly in China. It was found that the note types of the same population responding to different intruders were roughly the same, and that all the three populations had shared note types and their own unique note types to warn about the same intruder. Moreover, we found large differences in the acoustic parameters of shared common note types among populations. These findings provide valuable information to improve the collective understanding of the evolutionary mechanisms of alarm call systems in birds.

**Abstract:**

Geographic variability in acoustic signals has been documented in many bird species. However, geographic variations in alarm calls have been so far neglected despite their crucial role on reducing risk to group members and relatives. We analyzed the note types and acoustic parameters of Japanese tit (*Parus minor*) alarm calls to three types of intruders (a nest predator, an adult predator, and a harmless species) from three populations in China. Our results revealed that tits in the same population produce similar note types to different intruders, but the three populations only shared six note types and each population had unique note types. The frequency and duration parameters of three shared common note types were significantly different among populations. The three populations belong to the same species, thus they have shared note types. We suspect that the unique note types occurring in each population may be related to three potential reasons: founder effect, predation pressure, and vocal learning. The differences in acoustic parameters of common notes among populations may be a consequence of adaptations to their environments. We suggest that population differences in the note levels of bird alarm calls do exist.

## 1. Introduction

Bird acoustic signals are essential in territorial defense (i.e., territorial songs) and mate attraction (i.e., courtship songs) [1], as well as in anti-predator defense (i.e., alarm calls) and social communication (i.e., contact calls) [2,3]. However, many factors can influence geographic variation in acoustic signals, such as environment [4], signaler morphology [5], genetic drift [6], cultural drift [7], social pressures [8], or sexual selection [9]. Therefore, vocal communication in birds exhibits extensive regional differences [10,11,12].

To date, a large portion of what is known about geographic variation in bird acoustic signals has come from decades of study on songs [13]. Many studies have proven that geographic variation in songs can take place at several different levels, including notes, syllables, song types, or repertoires [14]. However, studies of geographic variation in call systems in birds are still relatively rare. Unlike song, which involves learning and therefore includes a cultural component in its vertical and horizontal transmission within oscine passerines (‘‘songbirds’’), bird calls have long been thought to be relatively impervious to experiential background [2]. Studying calls, therefore, introduces the opportunity to understand patterns of divergence in functionally and acoustically distinct signals that may be subject to different types of selection [15].

Geographic variation has been documented for bird calls [15,16,17,18], but most have focused on contact calls. Meanwhile, geographic variations in avian alarm calls have been thus far neglected. Many bird species can produce alarm calls after a predator has been detected [19]. Alarm calls may alert group members and kin of danger, call for assistance, or inform predators that they have been spotted and are no longer a threat [20]. Thus, alarm calls possess a crucial role in decreasing the likelihood of predation for conspecific [21] and heterospecific group members [22]. Brown and Farabaugh (1991) reported that alarm call types of Australian magpies (*Gymnorhina tibicen*) in two geographic populations showed significant geographic variation; some alarm call types occurred only in either the Australian population or in the New Zealand population, and some alarm call types occurred in both populations but varied in context between the two populations [23]. However, comparisons of alarm calls among the five populations of Thorn-tailed rayadito (*Aphrastura spinicauda*) in Chile showed that no differences were found among this type of vocalizations [11]. Researchers suggested that alarm calls are required to be understood by all members of the species across the entire distribution range, and thus, call differentiation is low [24]. There were so few existing studies on geographic variation of bird alarm calls, and the results of these studies were different. Therefore, it is necessary to conduct more comparative studies on alarm calls among populations to test whether there is geographic variation in alarm calls.

The alarm calls of chickadees, tits, and titmice (Family Paridae) is especially well-studied. Paridae species do not only transmit information in their alarm calls about the presence of a predator, but also about its threat level [25,26,27,28]. Information about a predator can be encoded by an increased call intensity, a variation in note number, note duration, or call type. In addition, the alarm call is comprised of distinct note types that follow rules of note ordering [29,30]. At present, independent studies on the geographical variation of alarm calls of parids are missing. One early study investigated the geographic variation of alarm calls in Siberian tits (*Poecile cinctus*) and found no variation in alarm calls of birds recorded in Norway as compared to birds recorded in eastern Siberia [31]. However, studies conducted on the geographic variation of chick-a-dee calls (intended to raise mild alarm and coordinate flock activities [32]) in Carolina chickadees (*Poecile carolinensis*) found that geographic variation existed in both note composition and uses of the chick-a-dee calls between Indiana and Tennessee populations [8,18].

The Japanese tit (*Parus minor*, Paridae) is a small songbird species distributed broadly across Northeast to South China. Across this range, there exists considerable variations in ecology, behavior, and life history. The wide geographical distribution of the Japanese tits makes it appropriate to study the variation of vocalizations across the species range. Previous studies showed that Japanese tits have a complex communication system that conveys information about predators, and this system contains multiple note types [33,34]. The main aim of the present study is to compare note types in alarm calls of three populations of Japanese tits (see details in Materials and Methods). According to previous studies, we hypothesized that there should be existing geographic variation among the note types in alarm calls of Japanese tits, but some note types will overlap partially among populations.

## 2. Materials and Methods

### 2.1. Study Area and Subjects

Field work was conducted from March to June in three sites within China: Zuojia Nature Reserve (126°0′–126°9′ E, 44°1′–44°6′ N) in Jilin Province, Dongzhai Nature Reserve (114°18′–114°30′ E, 31°28′–32°9′ N) in Henan Province, and in Diaoluoshan Nature Reserve (109°43′–110°3′ E, 18°43′–18°58′ N) in Hainan Province. In total, about 700 nest boxes were installed among the three geographical populations (about 400 in Jilin, about 150 in Henan, and about 150 in Hainan). The nest boxes were attached to trees about 2.5 m above the ground, facing in a random direction. Japanese tits are secondary-cavity nesters and prefer to select nest boxes as breeding sites in our study areas. We monitored the three populations nesting in nest boxes during the breeding seasons.

### 2.2. Dummy Experiments and Recordings

Previous studies revealed that tits could produce different alarm calls for different kinds of intruders [33,34]. From May to June, we used a nest predator common chipmunk *Tamias sibiricus*, an adult predator sparrowhawk *Accipiter nisus,* and a harmless species, Oriental turtle dove *Streptopelia orientalis,* to induce the alarm calls of tits, to collect as many note types of alarm calls as possible. During the nestling period, we placed one specimen on the nest box and then left quickly and hid about 15 m away when the parent birds were absent. Each nest received three dummy presentations in random order (n = 23 for Jilin between 2019–2021 (We identified different individuals through bands, and finally found that there were no duplicated individuals in the three-year experiment), n = 20 for Henan in 2021, and n = 13 for Hainan in 2021) to induce the tits’ alarm calls. In addition, two specimens for each species were randomly selected in each experiment [35].

The recording of alarm calls started when parent birds were observed within approximately 10 m of the specimen (included in recordings; see Audios S1–S9). Each recording lasted for 5 min [25]. A trial was terminated if no parent bird arrived within 30 min, and the next trial started at least 1 h later. All alarm calls of tits were recorded using a TASCAM DR-44WL recorder (Tascam, Tokyo, Japan), connected to a Sennheiser MKH P48 microphone (Sennheiser Electronic, Wedemark, Germany), with a sampling rate of 44.1 kHz and a 24-bit depth. All recordings were made during fine weather (e.g., no wind or rain) between 8:00 am and 6:00 pm.

### 2.3. Acoustic Analysis

The acoustic parameters of the notes were quantified using Avisoft SASLab Pro version 5.3.01 software (Avisoft Bioacoustics, Glienicke, Germany). Before alarm call analysis, noise <1 kHz was removed using audio filtering. The parameters used to generate a spectrogram were Blackman window, FFT-512, frame-100%, and overlap-87.5% (bandwidth 138 Hz, resolution 86 Hz). We included the first 20 alarm calls in each recording in these analyses. Alarm calls with fewer than 20 calls were all included for statistical analysis.

**Figure 1:**
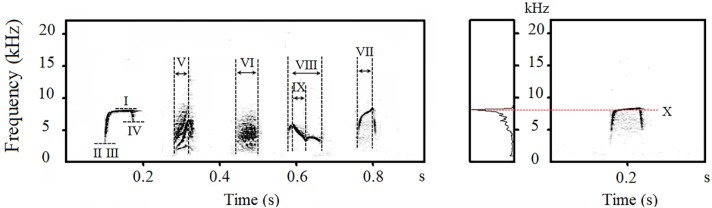
A sound spectrogram and spectra illustrating measurements of note acoustic parameters. Vertical lines indicate approximate boundaries for acoustic parameters. I: Maximum Frequency; II: Minimum Frequency; III: Start Frequency; IV: End Frequency; V: Ascending Duration; VI: Total Duration; VII: Maximum Frequency Duration; VIII: Minimum Frequency Duration; IX: Descending Duration; X: Peak Frequency.

Notes of Japanese tits’ alarm calls were classified into categories based on the visual similarity of the spectrograms [29,36]. A note was defined as any continuous trace on the spectrogram [37]. Based on the spectrogram, we divided the notes into D-type notes (D, M, and Hiss notes) and non-D-type notes, with the former possessing a harmonic-like structure and fuzzy edges. Therefore, we only measured two acoustic parameters of D-type notes: peak frequency and total duration. For non-D-type notes, we measured ten acoustic parameters: peak frequency, maximum frequency, minimum frequency, start frequency, end frequency, total duration, ascending duration, descending duration, maximum frequency duration, and minimum frequency duration (Figure 1, Explanation of acoustic parameters, see Table S1).

### 2.4. Note Descriptions

Common note-type descriptions are presented below.

A notes: These notes have an ascending arm, a peak, and a descending arm. The peak of the note remains stable for a small amount of time before descending. Usually, the ascending arm is similar to the descending arm in length, but occasionally short or even absent.

B notes: These notes have a long ascending arm beginning at a low frequency (about 2–2.5 kHz), which increases slowly at first and then rapidly to the peak frequency, then decreases to a frequency that is higher than the start frequency of the note. Harmonic-like structures can also be observed below and throughout B notes.

B_1_ notes: These notes possess the qualities of both A and B notes and appear to be an A note in transition to becoming a B note, thus forming a continuum of A→B notes. Their ascending arm and descending arm lengths are similar to the arms of the A notes. Their end frequency is always higher than start frequency, and the peak of the note is very pointed. These notes appear similar in total duration to B notes (usually less than 50 ms).

C notes: These notes have a short ascending arm and then decrease slowly to minimum frequency, followed by another short rising arm.

C_1_ notes: These notes have an extremely short ascending arm and then decrease slowly, with a small bandwidth and multiple harmonic-like structures above the maximum frequency band.

D notes: These notes have a harmonic-like structure, consisting of multiple frequency bands, with little frequency modulation. Occasionally noise flanks both the start and end of the note, leaving only the frequency bands in the middle portion of the note visible. These frequency bands have little frequency modulation, maintaining a constant frequency throughout the duration of the note. D notes are often longer in duration and lower in frequency than the other note types.

E notes: These notes are whistles, similar in structure to A notes. They have an ascending arm that usually begins at a high frequency, a short or no descending arm, and occasionally the ascending arm is shorter than the descending arm. Their total duration is longer than the duration of A notes (more than 120 ms).

I notes: I notes are tonal (i.e., no overtones or harmonic-like bands). These notes have multiple discrete cycles of ascending and descending frequency modulation throughout their duration. There is a very slight decrease in frequency from note start to note end.

G notes: These notes have a short ascending arm and then decrease slowly to the minimum frequency, and they have a peak in the middle of the descending arm. Some G notes also have a short ascending arm and a descending arm after the minimum frequency.

### 2.5. Statistical Data Analysis

To determine whether the notes of each type have been classified correctly according to spectrograms, Linear Discriminant Analysis (LDA) was performed on notes using their acoustic parameters, and the original types was set as grouping variable. Generalized linear mixed models (GLMMs, glmer in R package lme4) with a Poisson error structure and log-link function were used for the acoustic parameters of A, B, and D notes (the common shared note types in alarm calls among three populations), including population as a fixed effect and birds’ nests as random effects. Because two-group comparison after multiple comparisons will increase the probability of type I errors, we used FDR (false discovery rate) to adjust *p* values (p.adjust function in R package stats). All statistical analyses were conducted using R 4.1.1 (http://www.r-project.org, accessed on 15 November 2021).

## 3. Results

### 3.1. Note Classification

In Jilin-tits, there were twelve note types in alarm calls in response to common chipmunks, sparrowhawks, and Oriental turtle doves (Table 1). Ten of these note types were emitted in response to all three intruders. In Henan-tits, there were twelve note types in alarm calls in response to common chipmunks, thirteen note types in response to sparrowhawks, and eleven note types in response to Oriental turtle doves. Ten of these note types were emitted in response to all three intruders. In Hainan-tits, there were eleven note types in alarm calls in response to common chipmunks, thirteen note types in response to sparrowhawks, and ten note types in response to Oriental turtle doves. Eight of these note types were emitted in response to all three intruders. In response to common chipmunks, five note types (A, B, C, D, and E) were shared among three populations; Jilin-tits and Henan-tits had seven unique note types; Hainan-tits had six unique note types. In response to sparrowhawks, six note types (A, B, C, D, E, and G) were shared among three populations; Jilin-tits had six unique note types, Henan-tits and Hainan-tits had seven unique note types. In response to Oriental turtle doves, five note types (A, B, C, D, and G) were shared among three populations; Jilin-tits had seven unique note types, Henan-tits had six unique note types, and Hainan-tits had five unique note types.

**Figure 2:**
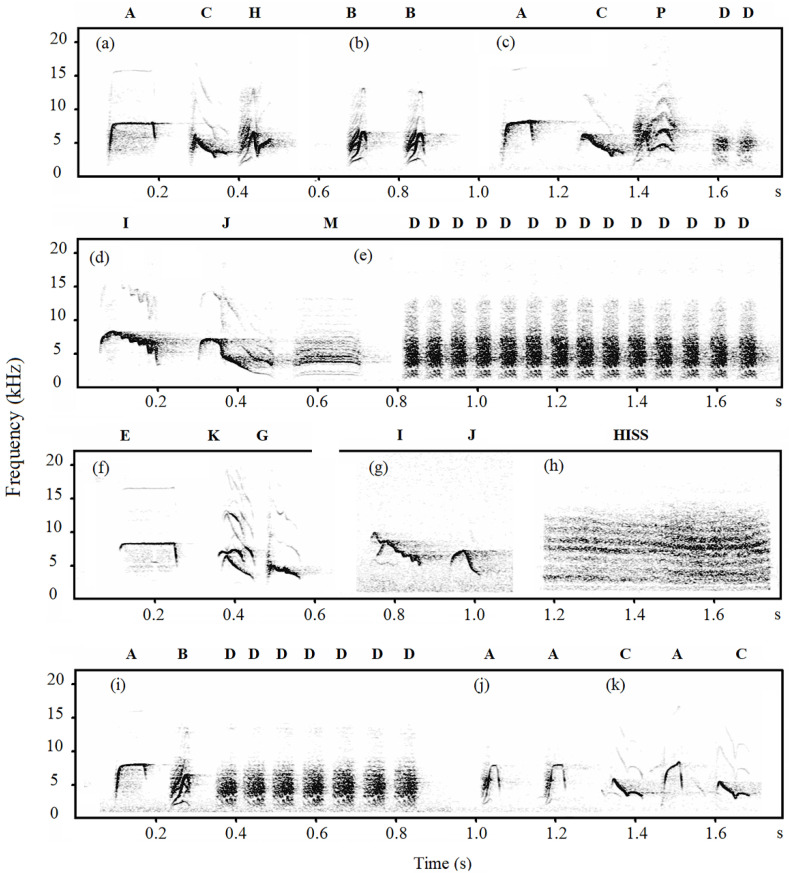
Spectrographic illustration of alarm calls of Jilin-tits (capital letters are notes and lowercase letters are calls).

**Figure 3:**
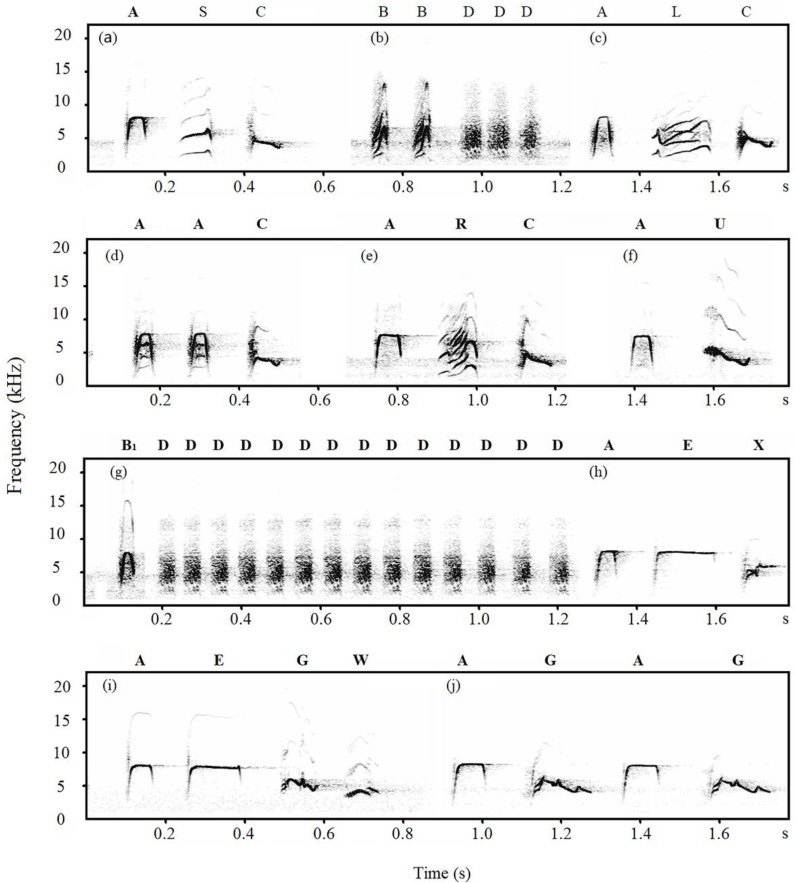
Spectrographic illustration of alarm calls of Henan-tits (capital letters are notes and lowercase letters are calls).

**Figure 4:**
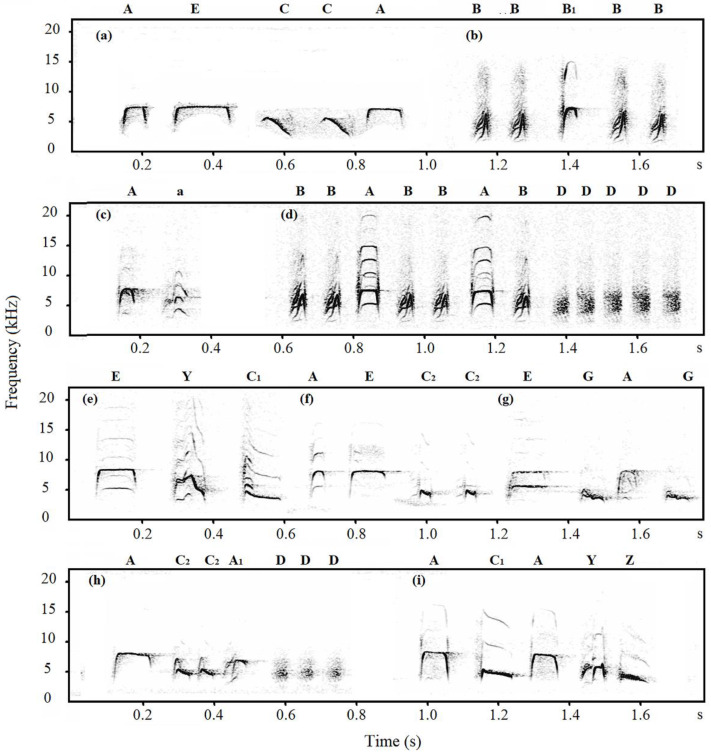
Spectrographic illustration of alarm calls of Hainan-tits (capital letters are notes and lowercase letters are calls).

In total, thirteen note types were identified in alarm calls of Jilin-tits, Henan-tits and Hainan-tits (Figure 2, Figure 3 and Figure 4). In Jilin-tit alarm calls, the common note types were A, B, D, and I, which were present in more than 50% of the nests’ alarm calls. In Henan-tit alarm calls, the common note types were A, B, D, and E. In Hainan-tit alarm calls, the common note types were A, B, C_1_, and D. In addition, note types A, B, C, D, E, and G occurred in all three populations, and B_1_ occurred in both the Henan population and the Hainan population. Those shared note types share similar spectrogram structures.

### 3.2. Note Discriminant Analysis

The LDA results indicate that Japanese tit note types are distinct. For Jilin-tit notes, LDA correctly classified 93.9% of non-D-type notes on the basis of differences in 10 acoustic parameters. The first two LD functions accounted for 79.1% of the total variance explained. Likewise, LDA correctly classified 98.1% of D-type notes on the basis of differences in two acoustic parameters. The first two LD functions accounted for 100% of the total variance explained. For Henan-tit notes, LDA correctly classified 92.8% of non-D-type notes on the basis of differences in 10 acoustic parameters. The first two LD functions accounted for 73.7% of the total variance explained. For Hainan-tit note types, LDA correctly classified 90.1% of non-D-type notes on the basis of differences in 10 acoustic parameters. The first two LD functions accounted for 78.70% of the total variance explained. Henan and Hainan tits have only one D-type note (i.e., D notes); no discriminant analysis was carried out.

The results of LDA revealed that more than 90% of notes in each population were correctly classified; these results supported the note classifications of the spectrograms.

### 3.3. Comparison of Shared Note Types

#### 3.3.1. Comparison of A Notes

The results showed that the peak frequency of A notes do not differ significantly among populations (GLMMs, χ^2^ = 2.55, df = 2, *p* = 0.279), but maximum frequency (χ^2^ = 13.54, df = 2, *p* = 0.001), minimum frequency (χ^2^ = 11.51, df = 2, *p* = 0.003), start frequency (χ^2^ = 12.21, df = 2, *p* = 0.002), and end frequency (χ^2^ = 8.82, df = 2, *p* = 0.012) all differ significantly among populations. The maximum frequency of Hainan-tits is significantly lower than those of Jilin-tits or Henan-tits, but there was no significant difference between Jilin-tits and Henan-tits. Both the minimum frequency and start frequency of Henan-tits are significantly lower than Jilin-tits or Hainan-tits, but no significant difference was found between Jilin-tits and Hainan-tits. The end frequency of Jilin-tits is significantly higher than those of Henan-tits and Hainan-tits, but there was no significant difference found between Henan-tits and Hainan-tits (Table 2).

Similarly, it was discovered that total duration (χ^2^ = 28.10, df = 2, *p* < 0.001), ascending duration (χ^2^ = 24.52, df = 2, *p* < 0.001), descending duration (χ^2^ = 31.64, df = 2, *p* < 0.001), maximum frequency duration (χ^2^ = 15.08, df = 2, *p* < 0.001), and minimum frequency duration (χ^2^ = 10.60, df = 2, *p* = 0.005) of A notes all differed significantly among the populations. Total duration, ascending duration, and descending duration differ significantly between Jilin-tits and Henan-tits or Hainan-tits. A notes of Jilin-tits had significantly longer total duration, shorter ascending duration, and descending duration as compared to those of the other two populations, but there was no difference between Henan-tits and Hainan-tits. The maximum frequency of Jilin-tits was significantly longer than that of Hainan-tits, but no difference was found between Henan-tits and Jilin-tits or Hainan-tits. The minimum frequency duration differed significantly between Jilin-tits and Henan-tits, and was shorter in Jilin-tits. There was no significant difference in minimum frequency between Hainan-tits and Jilin-tits or Henan-tits (Table 2).

#### 3.3.2. Comparison of B Notes

With the exception of peak frequency (χ^2^ = 1.80, df = 2, *p* = 0.406), we found that maximum frequency (χ^2^ = 11.86, df = 2, *p* = 0.003), minimum frequency (χ^2^ = 23.23, df = 2, *p* < 0.001), start frequency (χ^2^ = 22.83, df = 2, *p* < 0.001), and end frequency (χ^2^ = 90.10, df = 2, *p* < 0.001) of B notes differed significantly between populations. The maximum frequency of Jilin-tits is significantly lower than those of Henan-tits and Hainan-tits, but no significant difference was found between Henan-tits and Hainan-tits. Both the minimum frequency and the start frequency of Jilin-tits were significantly lower than those of the Henan-tits and Hainan-tits, and those of Henan-tits were significantly lower than that of Hainan-tits. The end frequency of Jilin-tits was significantly higher than those of Henan-tits and Hainan-tits, and the end frequency of Henan-tits was significantly higher than that of Hainan-tits (Table 3).

Similarly, total duration (χ^2^ = 15.75, df = 2, *p* < 0.001), ascending duration (χ^2^ = 30.75, df = 2, *p* < 0.001), and maximum frequency duration (χ^2^ = 57.15, df = 2, *p* < 0.001) of B notes all differed significantly among populations, except descending duration (χ^2^ = 2.91, df = 2, *p* = 0.234). The total duration of Jilin-tits was significantly longer than those of Henan-tits and Hainan-tits, but no significant difference was found between Henan-tits and Hainan-tits. The ascending duration of Jilin-tits was significantly longer than those of Henan-tits and Hainan-tits, and the ascending duration of Henan-tits was significantly longer than that of Hainan-tits. The maximum frequency duration of Jilin-tits was significantly shorter than those of Henan-tits and Hainan-tits, and the maximum frequency duration of Henan-tits was significantly shorter than that of Hainan-tits (Table 3).

#### 3.3.3. Comparison of D Notes

The total duration of D notes was significantly different among the three populations (χ^2^ = 13.69, df = 2, *p* = 0.001), but the peak frequency did not differ among populations (χ^2^ = 0.12, df = 2, *p* = 0.944). The total duration of Jilin-tits was significantly shorter than that of Hainan-tits (adjust *p* < 0.001, Jilin-tits: 46.16 ± 0.20 ms, Hainan-tits: 51.27 ± 0.36 ms), but no difference was found between Jilin-tits and Henan-tits or Henan-tits and Hainan-tits (adjust *p* > 0.051 for both).

## 4. Discussion

Birds can encode threatening information about predators by using different call types [21,26,38], note type combinations [18,33,34], calling rates [39], the number of notes per call [33,40], and the compositional syntax of an alarm call [41]. In this study, the note types of the same population responding to three intruders were roughly the same (10/13, 10/13, 8/13 for Jilin-tit, Henan-tit and Hainan-tit populations, respectively), but only a few note types were particular to a special intruder (Table 1). Our results suggest that all three populations of Japanese tits use a limited number of note types to transmit information regarding threats. Therefore, we speculate that the encoding mechanisms of Japanese tit alarm calls should not be based on particular note types, but rather should adopt mechanisms such as note type combinations or number of notes per call [33,34].

Geographic variation in the note-level of bird songs has been reported [42,43]. For instance, singing honeyeater (*Lichenostomus virescens*) populations on the mainland possess a large diversity of notes, whereas, on Rottnest, the population pool of notes is greatly reduced, and possesses few notes that are structurally similar to mainland ones [44]. In the present study, we found 13 note types in alarm calls across three populations of Japanese tits. Among those note types, six note types were shared among the three populations, and one note type was shared between the Henan and Hainan populations. In addition, there were five or six shared note types in the alarm calls of the three populations responding to the same intruder. The three populations in our study belong to the same species, explaining their similar note types.

However, the Jilin population had seven unique note types, and both Henan and Hainan populations had six unique note types. Furthermore, there were 5–7 unique note types in alarm calls of different populations responding to the same intruder. All three populations used similar shared note types and their own unique note types to transmit threat information in alarm calls. Here, we suggest there may be three possible reasons for unique note types in each population. First, the founder effect could result in the loss of some note types or the formation of new note types over time. When a new population is established by a few individuals, the signal characteristics of the population largely depend on its founders [45]. Japanese tits are non-migratory, and the present distribution of these tits is similar to their distribution during the LGM (Last Glacial Maximum) [46]. It seems likely that the three populations have been separated for at least 20,000 years, and possibly for much longer. Second, the predation pressure difference among the three populations might be involved in the evolution of bird alarm calls [19,47]. Predator species vary over space and may promote divergence in signals conveying broad information [47]. Finally, the differences in alarm calls may be closely correlated to vocal learning [48]. For instance, greater racket-tailed drongos (*Dicrurus paradiseus*) incorporated the alarm-associated notes of other species in their alarm calls [49]. In this study, the community composition of the three sites was different, which may promote the three populations forming different note types. Different vocalization levels may be affected by different factors and result in different patterns of geographic variation (Tracy and Baker 1999). Signaler morphology may be related to the elaboration of original notes rather than the occurrence of new note types [43]. In conclusion, consistent with bird songs, our results indicated that alarm calls also differ in note-level among different geographic populations.

Additionally, we found population differences among the acoustic parameters of the shared common note types: A, B, and D notes. Avian species had vocal plasticity, which would appear to be advantageous for birds to modify parameters of their calls to adapt to social and physical environments [50,51]. For example, great tits (*Parus major*) could increase the minimum frequency of their songs to avoid being drowned out by the local background noise [52]. In addition, raptors can locate birds through their acoustic signals [53]; for example, the best hearing range for sparrowhawks is 1–4 kHz [54]. In this study, the number of predator species is highest in Hainan, and the majority of the population of Hainan-tits live along the road in forests with many vehicles passing, which may result in Hainan-tits increasing their minimum frequencies of A and B notes. Meanwhile, Hainan-tits living in tropical rainforests at low latitudes with dense vegetation and attenuation of higher frequencies over distance is more pronounced in denser habitats. Therefore, Hainan-tits appropriately reduce maximum frequency to avoid the frequency-dependent attenuation and acquisition of signals by potential raptors [4,51,55]. Vegetation density and predator species decrease with increasing latitude. Jilin-tits live in high-latitude areas with broad-leaved forests, so the frequency of notes usually differs significantly from that of the Hainan-tits. Hence, we suggested tits could adjust their notes’ frequency to adapt to local habitat structure and predator pressure.

Research has demonstrated that signalers might extend the duration of individual notes within the signal to increase signal detectability by conspecifics [56]. Furthermore, studies discovered that some non-D-type notes of black-capped chickadees (*Poecile atricapilla*) could convey information about close-range predators [57], while D notes of birds could recruit conspecifics and heterospecifics to mob predators [41]. The population density of Henan-tits and Hainan-tits was smaller than that of Jilin-tits, so threat information transmission in long range to potential receivers might be not effective in Henan-tits and Hainan-tits. Therefore, the total durations of A and B notes in Henan-tits and Hainan-tits were shorter than that in Jilin-tits. Hainan-tits pronounced a longer total duration of D notes than Jilin tits, which perhaps helped them to recruit long-distance members to harass or mob a predator. In summary, we speculated that the population differences in duration and frequency parameters of A, B, and D notes may be the consequences of the tits’ adaptation to the environments and predation pressures.

## 5. Conclusions

In this study, we found population differences in the note types of alarm calls in Japanese tits. The three populations shared six note types, and each population also had unique note types. The frequency and duration parameters of common shared note types (i.e., A, B, and D notes) were significantly different among populations. However, different call levels may be affected by different factors and result in different patterns of geographic variation. Since our study only focused on the note level, we suggest further analysis of the other levels in the alarm call hierarchy in the future.

## Figures and Tables

**Table 1:** Note types in alarm calls of three populations of Japanese tits in response to common chipmunks, sparrowhawks, and Oriental turtle doves.

Population	Specimen	Note Types
Jilin-tits	Chipmunk	A, B, C, D, E, G, H, HISS, I, J, P, Q
	Sparrowhawk	A, B, C, D, E, G, H, I, J, K, M, P
	Dove	A, B, C, D, E, G, H, I, J, K, M, P
Henan-tits	Chipmunk	A, B, B_1_, C, D, E, G, L, R, U, W, X
	Sparrowhawk	A, B, B_1_, C, D, E, G, L, R, S, U, W, X
	Dove	A, B, B_1_, C, D, E, G, L, S, W, X
Hainan-tits	Chipmunk	a, A, B, B_1_, C, C_1_, C_2_, D, E, Y, Z
	Sparrowhawk	a, A, A_1_, B, B_1_, C, C_1_, C_2_, D, E, G, Y, Z
	Dove	a, A, A_1_, B, B_1_, C_1_, C_2_, D, G, Y

**Table 2:** The results of comparisons of the acoustic parameters of A notes in alarm calls of Japanese tits.

Acoustic Parameter	Population	Mean ± SE	*Post-Hoc p* Value	
			Henan	Hainan
Maximum frequency (Hz)	Jilin	8220.64 ± 10.44	0.704	0.003 **
	Henan	8217.81 ± 8.41		0.002 **
	Hainan	7951.81 ± 13.92		
Minimum frequency (Hz)	Jilin	4315.34 ± 41.68	0.019 *	0.323
	Henan	3890.58 ± 29.64		0.004 **
	Hainan	4505.45 ± 34.11		
Start frequency (Hz)	Jilin	4408.91 ± 47.11	0.015 *	0.302
	Henan	3902.82 ± 30.45		0.003 **
	Hainan	4614.76 ± 37.20		
End frequency (Hz)	Jilin	5831.46 ± 39.23	0.013 *	0.071 *
	Henan	5068.70 ± 35.63		0.482
	Hainan	5185.46 ± 35.99		
Total duration (ms)	Jilin	84.91 ± 0.79	<0.001 **	<0.001 **
	Henan	61.26 ± 0.63		0.241
	Hainan	69.31 ± 0.77		
Ascending duration (ms)	Jilin	16.09 ± 0.17	<0.001 **	<0.001 **
	Henan	18.00 ± 0.12		0.292
	Hainan	18.57 ± 0.13		
Descending duration (ms)	Jilin	11.44 ± 0.13	<0.001 **	<0.001 **
	Henan	13.21 ± 0.16		0.169
	Hainan	13.97 ± 0.14		
Maximum frequency duration (ms)	Jilin	43.15 ± 1.10	0.062	<0.001 **
	Henan	29.84 ± 0.58		0.062
	Hainan	25.79 ± 0.53		
Minimum frequency duration (ms)	Jilin	6.74 ± 0.89	0.006 **	0.535
	Henan	1.22 ± 0.35		0.053
	Hainan	12.15 ± 0.96		

Note: * *p* < 0.05, ** *p* < 0.01. *p* values were adjusted by FDR.

**Table 3:** The results of comparisons of the acoustic parameters of B notes in the alarm calls of Japanese tits.

Acoustic Parameter	Population	Mean ± SE	*Post-Hoc p* Value	
			Henan	Hainan
Maximum frequency (Hz)	Jilin	6552.83 ± 17.69	0.006 **	0.006 **
	Henan	6880.25 ± 17.61		0.837
	Hainan	6971.67 ± 19.72		
Minimum frequency (Hz)	Jilin	2228.19 ± 18.31	0.022 *	<0.001 **
	Henan	2383.02 ± 12.70		0.015 *
	Hainan	2561.99 ± 12.15		
Start frequency (Hz)	Jilin	2239.36 ± 19.79	0.041 *	<0.001 **
	Henan	2382.42 ± 12.75		0.008 **
	Hainan	2564.17 ± 12.45		
End frequency (Hz)	Jilin	5210.94 ± 27.90	<0.001 **	<0.001 **
	Henan	4459.36 ± 34.01		<0.001 **
	Hainan	3635.24 ± 22.41		
Total duration (ms)	Jilin	48.42 ± 0.22	0.003 **	<0.001 **
	Henan	46.02 ± 0.25		0.628
	Hainan	45.44 ± 0.17		
Ascending duration (ms)	Jilin	25.66 ± 0.27	0.021 *	<0.001 **
	Henan	27.77 ± 0.28		0.002 **
	Hainan	31.87 ± 0.20		
Maximum frequency duration (ms)	Jilin	33.56 ± 0.27	0.001 **	<0.001 **
	Henan	30.11 ± 0.24		<0.001 **
	Hainan	25.59 ± 0.17		

Note: * *p* < 0.05, ** *p* < 0.01. *p* values were adjusted by FDR.

## Data Availability

The data presented in this study are available on request from the corresponding author.

## Supplementary Materials

The following supporting information can be downloaded at: https://www.mdpi.com/article/10.3390/ani12182342/s1, Table S1: Explanation of acoustic parameters; Audio S1: Alarm calls of Hainan-tits in response to chipmunks; Audio S2: Alarm calls of Hainan-tits in response to sparrowhawk; Audio S3: Alarm calls of Hainan-tits in response to dove; Audio S4: Alarm calls of Henan-tits in response to chipmunks; Audio S5: Alarm calls of Henan-tits in response to sparrowhawk; Audio S6: Alarm calls of Henan-tits in response to dove; Audio S7: Alarm calls of Jilin-tits in response to chipmunks; Audio S8: Alarm calls of Jilin-tits in response to sparrowhawk; Audio S9: Alarm calls of Jilin-tits in response to dove.

## References

[1] Catchpole C.K. (1987). Bird song, sexual selection and female choice. Trends Ecol. Evol..

[2] Marler P.R., Marler P.R., Slabbekoorn H. (2004). Bird calls: A cornucopia for communication. Nature’s Music: The Science of Birdsong.

[3] Magrath R.D., Pitcher B.J., Gardner J.L. (2007). A mutual understanding? Interspecific responses by birds to each other’s aerial alarm calls. Behav. Ecol..

[4] Nicholls J.A., Goldizen A.W. (2006). Habitat type and density influence vocal signal design in satin bowerbirds. J. Anim. Ecol..

[5] Ballentine B. (2006). Morphological adaptation influences the evolution of a mating signal. Evolution.

[6] Irwin D.E., Thimgan M.P., Irwin J.H. (2008). Call divergence is correlated with geographic and genetic distance in greenish warblers (*Phylloscopus trochiloides*): A strong role for stochasticity in signal evolution?. J. Evol. Biol..

[7] Beecher M.D., Brenowitz E.A. (2005). Functional aspects of song learning in songbirds. Trends Ecol. Evol..

[8] Freeberg T.M. (2012). Geographic variation in note composition and use of chick-a-dee calls of Carolina Chickadees (*Poecile carolinensis*). Ethology.

[9] Fuisz T.I., de Kort S.R. (2007). Habitat-dependent call divergence in the common cuckoo: Is it a potential signal for assortative mating?. Proc. R. Soc. B-Biol. Sci..

[10] Irwin D.E. (2000). Song variation in an avian ring species. Evolution.

[11] Ippi S., Vasquez R.A., van Dongen W.F.D., Lazzoni I. (2011). Geographical variation in the vocalizations of the suboscine Thorn-tailed Rayadito *Aphrastura spinicauda*. Ibis.

[12] Wei C., Jia C., Dong L., Wang D., Xia C., Zhang Y., Liang W. (2015). Geographic variation in the calls of the Common Cuckoo (*Cuculus canorus*): Isolation by distance and divergence among subspecies. J. Ornithol..

[13] Podos J., Warren P.S. (2007). The evolution of geographic variation in birdsong. Adv. Study Behav..

[14] Slater P.J.B. (1989). Bird song learning: Causes and consequences. Ethol. Ecol. Evol..

[15] Luttrell S.A.M., Lohr B. (2018). Geographic variation in call structure, likelihood, and call-song associations across subspecies boundaries, migratory patterns, and habitat types in the Marsh Wren (*Cistothorus palustris*). Auk.

[16] Bradbury J.W., Cortopassi K.A., Clemmons J.R. (2001). Geographical variation in the contact calls of orange-fronted Parakeets. Auk.

[17] Naguib M., Hammerschmidt K., Wirth J. (2001). Microgeographic variation, habitat effects and individual signature cues in calls of chiffchaffs *Phylloscopus collybita canarensis*. Ethology.

[18] Freeberg T.M. (2008). Complexity in the chick-a-dee call of Carolina Chickadees (*Poecile carolinensis*): Associations of context and signaler behavior to call structure. Auk.

[19] Gill S.A., Bierema A.M.K. (2013). On the meaning of alarm calls: A review of functional reference in avian alarm calling. Ethology.

[20] Griesser M. (2009). Mobbing calls signal predator category in a kin group-living bird species. Proc. R. Soc. B-Biol. Sci..

[21] Templeton C.N., Greene E., Davis K. (2005). Allometry of alarm calls: Black-capped chickadees encode information about predator size. Science.

[22] Templeton C.N., Greene E. (2007). Nuthatches eavesdrop on variations in heterospecific chickadee mobbing alarm calls. Proc. Natl. Acad. Sci. USA.

[23] Brown E.D., Farabaugh S.M. (1991). Macrogeographic variation in alarm calls of the Australian Magpie *Gymnorhina-Tibicen*. Bird Behav..

[24] Habel J.C., Husemann M., Ulrich W. (2018). Evolution of contact and alarm calls in the Kenyan endemic Hinde’s babbler (Aves: Passeriformes). BMC Evol. Biol..

[25] Courter J.R., Ritchison G. (2010). Alarm calls of tufted titmice convey information about predator size and threat. Behav. Ecol..

[26] Suzuki T.N. (2015). Assessment of predation risk through referential communication in incubating birds. Sci. Rep..

[27] Dutour M., Lena J.P., Lengagne T. (2016). Mobbing behaviour varies according to predator dangerousness and occurrence. Anim. Behav..

[28] Carlson N.V., Healy S.D., Templeton C.N. (2017). A comparative study of how British tits encode predator threat in their mobbing calls. Anim. Behav..

[29] Bloomfield L.L., Phillmore L.S., Weisman R.G., Sturdy C.B. (2005). Note types and coding in parid vocalizations. III: The chick-a-dee call of the Carolina chickadee (*Poecile carolinensis*). Can. J. Zool..

[30] Freeberg T.M., Lucas J.R., Krams I. (2012). The complex call of the Carolina Chickadee. What can the chick-a-dee call teach us about communication and language?. Am. Sci..

[31] Haftorn S., Hailman J.P. (1997). Do the Siberian tits *Parus cinctus* in Scandinavia and Siberia speak the same language?. Bioacoustics.

[32] Hailman J.P., Ficken M.S., Ficken R.W. (1985). The ‘chick-a-dee’ calls of *Parus atricapillus*: A recombinant system of animal communication compared with written English. Semiotica.

[33] Yu J.P., Xing X.Y., Jiang Y.L., Liang W., Wang H.T., Moller A.P. (2017). Alarm call-based discrimination between common cuckoo and Eurasian sparrowhawk in a Chinese population of great tits. Ethology.

[34] Suzuki T.N. (2014). Communication about predator type by a bird using discrete, graded and combinatorial variation in alarm calls. Anim. Behav..

[35] Yu J., Zhang L., Yi G., Zhang K., Yao J., Fang J., Shen C., Wang H. (2021). Plastering mud around the entrance hole affects the estimation of threat levels from nest predators in Eurasian Nuthatches. Avian Res..

[36] Bloomfield L.L., Charrier I., Sturdy C.B. (2004). Note types and coding in parid vocalizations. II: The chick-a-dee call of the mountain chickadee (*Poecile gambeli*). Can. J. Zool..

[37] Baptista L.F. (1977). Geographic variation in song and dialects of puget sound white-crowned sparrow. Condor.

[38] Suzuki T.N., Ueda K. (2013). Mobbing calls of Japanese tits signal predator type: Field observations of natural predator encounters. Wilson J. Ornithol..

[39] Wilson D.R., Mennill D.J. (2011). Duty cycle, not signal structure, explains conspecific and heterospecific responses to the calls of black-capped chickadees (*Poecile atricapillus*). Behav. Ecol..

[40] Leavesley A.J., Magrath R.D. (2005). Communicating about danger: Urgency alarm calling in a bird. Anim. Behav..

[41] Suzuki T.N. (2016). Referential calls coordinate multi-species mobbing in a forest bird community. J. Ethol..

[42] Tu H.W., Severinghaus L.L. (2004). Geographic variation of the highly complex Hwamei (*Garrulax canorus*) songs. Zool. Stud..

[43] Bolus R.T. (2014). Geographic variation in songs of the Common Yellowthroat. Auk.

[44] Baker M.C., Baker M.S.A., Tilghman L.M. (2006). Differing effects of isolation on evolution of bird songs: Examples from an island-mainland comparison of three species. Biol. J. Linn. Soc..

[45] Lack D., Southern H.N. (1949). Birds on Tenerife. Ibis.

[46] Zhao N., Dai C., Wang W., Zhang R., Qu Y., Song G., Chen K., Yang X., Zou F., Lei F. (2012). Pleistocene climate changes shaped the divergence and demography of Asian populations of the great tit *Parus major*: Evidence from phylogeographic analysis and ecological niche models. J. Avian Biol..

[47] Wheatcroft D., Price T.D. (2015). Rates of signal evolution are associated with the nature of interspecific communication. Behav. Ecol..

[48] Greenlaw J.S., Shackelford C.E., Brown R.E. (1998). Call mimicry by eastern towhees and its significance in relation to auditory learning. Wilson Bull..

[49] Goodale E., Kotagama S.W. (2006). Context-dependent vocal mimicry in a passerine bird. Proc. R. Soc. B-Biol. Sci..

[50] Freeberg T.M., Lucas J.R., Clucas B. (2003). Variation in chick-a-dee calls of a Carolina Chickadee population, *Poecile carolinensis*: Identity and redundancy within note types. J. Acoust. Soc. Am..

[51] Proppe D.S., Bloomfield L.L., Sturdy C.B. (2010). Acoustic transmission of the chick-a-dee call of the Black-capped Chickadee (*Poecile atricapillus*): Forest structure and note function. Can. J. Zool..

[52] Slabbekoorn H., den Boer-Visser A. (2006). Cities change the songs of birds. Curr. Biol..

[53] Krams I. (2001). Communication in crested tits and the risk of predation. Anim. Behav..

[54] Krama T., Krams I., Igaune K. (2008). Effects of cover on loud trill-call and soft seet-call use in the crested tit *Parus Cristatus*. Ethology.

[55] Seddon N. (2005). Ecological adaptation and species recognition drives vocal evolution in neotropical suboscine birds. Evolution.

[56] Pohl N.U., Slabbekoorn H., Neubauer H., Heil P., Klump G.M., Langemann U. (2013). Why longer song elements are easier to detect: Threshold level-duration functions in the Great Tit and comparison with human data. J. Comp. Physiol. A-Neuroethol. Sens. Neural Behav. Physiol..

[57] Baker M.C., Becker A.M. (2002). Mobbing calls of Black-capped Chickadees: Effects of urgency on call production. Wilson Bull..

