# Supplementary material for: Geographic Variation in Note Types of Alarm Calls in Japanese Tits (Parus minor)
Source: Animals (Basel). 2022 Sep 8;12(18):2342. doi: 10.3390/ani12182342 (PMC9495063; doi:10.3390/ani12182342)
Supplement: Supplementary file 1 [file animals-12-02342-s001.zip › Table S1,Explanation of acoustic parameters..pdf]

Table S1. Explanation of acoustic parameters

Acoustic parameter	Explanation
Total Duration	Duration of note from start to end
Ascending Duration	Duration from the start to the end of the first ascending arm of each note
Descending Duration	Duration from the start to the end of the first descending arm of each note
Maximum Frequency Duration	Duration from the start of note to the location of the maximum frequency within each note
Minimum Frequency Duration	Duration from the start of note to the location of the minimum frequency within each note
Peak Frequency	Frequency with greatest amplitude
Maximum Frequency	Frequency of the maximum amplitude of the spectrum
Minimum Frequency	Frequency of the minimum amplitude of the spectrum
Start Frequency	Frequency at the start of each note
End Frequency	Frequency at the end of each note
